# Sorption of Polar Sorbates NH_3_, H_2_O, SO_2_ and CO_2_ on Selected Inorganic Materials

**DOI:** 10.3390/ma16134853

**Published:** 2023-07-06

**Authors:** Katarzyna Zarębska, Mikihiro Nomura, Marta Wolczko, Jakub Szczurowski, Bartłomiej Pawlak, Paweł Baran

**Affiliations:** 1 Faculty of Energy and Fuels, AGH University of Science and Technology, Al. Mickiewicza 30, 30-059 Krakow, Poland; wolczko@agh.edu.pl (M.W.); szczurow@agh.edu.pl (J.S.); bpawlak@agh.edu.pl (B.P.); baranp@agh.edu.pl (P.B.); 2 Department of Applied Chemistry, Shibaura Institute of Technology, 3-7-5 Toyosu, Koto-ku, Tokyo 135-8548, Japan; lscathy@shibaura-it.ac.jp

**Keywords:** sorption, polar sorbates, hydrogen bond, molecular sieves

## Abstract

In this paper, the sorption of NH_3_, H_2_O, SO_2_ and CO_2_ was tested for several selected inorganic materials. The tests were performed on samples belonging to two topologies of materials, faujasite (FAU) and framework-type MFI, the structures of which differ in pore size and connectivity. All sorbates are important in terms of reducing their emissions to the environment. They have different chemical nature: basic, alkaline, and acidic. They are all polar in structure and composition and two of them (ammonia and water vapor) can form hydrogen bonds. These differences result in different interactions with the surface of the adsorbents. This paper presents experimental data and proposes a mathematical description of the sorption process. The best fit of the experimental data was obtained for the Toth and GAB models. The studies showed that among the selected samples, faujasite has the best sorption capacity for ammonia and water vapor, while the best sorbent for sulfur dioxide is the MFI framework type. These materials behave like molecular sieves and can be used for quite selective adsorption of relevant gases. In addition, modification of the faujasite with organic silane resulted in a drastic reduction in the surface area of the sorbent, resulting in significantly lower sorption capacities for gases.

## 1. Introduction

Capture of NH_3_, H_2_O, SO_2_, and CO_2_ is an important and challenging topic in industry and environmental protection due to increasing standards of emission.

Ammonia is one of the most toxic and corrosive gases that is used intensively and produced on a large scale in industrial processes. The annual production of ammonia can be measured in millions of tons, making it one of the most widely used chemicals. The high toxicity of this chemical compound is observed even at low concentrations, and in addition, it is highly corrosive, making it difficult to study. Furthermore, this compound is used in fertilizer production, as a refrigerant that contributes to eutrophication and it strongly influences the greenhouse effect. Ammonia is also an undesirable odor gas, and its removal is an important part of large-scale animal husbandry.

The choice of water as a sorbent is dictated by the fact that water vapor is the gas that causes practically half of the greenhouse effect on earth. From an experimental point of view, water vapor is an excellent probe for assessing the chemical nature of the sorbent surface. Due to its polar nature and ability to form hydrogen bonds, it can form clusters and interact with individual surface groups.

Sulfur dioxide is a by-product of the burning of waste in domestic furnaces and is also a significant component of the smog inhaled by residents of large urban areas. It is a colorless, non-flammable gas with a strongly pungent, suffocating odor. It is widely used in the food industry (E220). It acts primarily as a preservative and antioxidant. Sulfur dioxide and its derivatives are recognized as a potent allergen, and along with peanuts, hazelnuts, and shellfish, it is on the EU list of allergens. Removal of low-concentration sulfur dioxide from gas mixture is of great significance in gas purification, but the selective recognition of SO_2_ from carbon dioxide (CO_2_) still remains a great challenge with low separation selectivity.

Carbon dioxide is now recognized as the most important and widespread greenhouse gas. Despite regulations to reduce its emissions, and available technologies to remove it, new ways to decarbonize it are still being sought. Furthermore, as a chemical compound that possesses a quadrupole dipole moment, it plays an important role in understanding sorption mechanisms. In addition to being used to study the porous texture of metaparticles, it complements studies of microporosity and surface character.

Current knowledge shows that adsorption processes play an important role in both the capture and regeneration of materials used for gas removal. Zeolites, MOFs, and carbon materials, with sorption properties that can be shaped in a wide range, are used for this purpose. MOFs and zeolites have a higher affinity for polar sorbates compared to activated carbon. However, these materials can be structurally degraded, for example, by ammonia [1,2,3]. Therefore, zeolites are an interesting alternative due to their good adsorption properties and lower production costs.

Zeolites are microporous crystalline aluminosilicates widely used as inorganic adsorbents because of their ability to adsorb polar compounds. Zeolites exist in both natural and synthetic form, characterized by different zeolites in Si/Al ratios and a pore size in the range of 0.3–1 nm. They have a high internal surface area available for sorption due to channels that evenly penetrate the entire volume of the structure [4,5].

These materials have been used as adsorbents for several decades, finding applications in numerous fields [5,6,7]. Low-cost sorption may provide an advantage over traditional separation and purification processes, such as distillation, extraction, or absorption.

Natural zeolites have been used in the upgrading of natural gas to remove N_2_, CO_2_, H_2_O, and sulfur contaminants, such as H_2_S and mercaptans. Other uses include the purification of hydrocarbons, the purification of hydrogen, and the prepurification of air from CO_2_, NO_x_, SO_x_, etc. Moreover, modification of their adsorptive properties via ion exchange, thermal treatment, and structural changes could improve the separation potential [5].

Zeolites can be used as an adsorbent for the removal of water vapor from natural gas or other hydrocarbon streams [8], NH_3_ from coal gasification products [9] or for the removal of NH_4_^+^ ions from an aqueous solution [10]. The removal of SO_2_ from air, flue gases, and hydrocarbon feedstreams is also an important separation in the oil, gas, and chemical process industries and, of course, for environmental purposes [11,12,13,14,15].

Due to the toxicity and high reactivity of both NH_3_ and SO_2_, there are only a few studies on the adsorption of these gases in the literature [16,17,18,19]. In these studies, zeolites containing cations showed stronger ammonia adsorption than pure silica structures at low pressure [17,18].

However, the hydrophobic nature of pure silica zeolites makes them better candidates for processes where water competition must be avoided. Jaramillo and Chandro’s [19] investigated the sorption capacity of ammonia, carbon dioxide, and water vapor on zeolite 4A and showed that the experimental data of NH_3_ and CO_2_ isotherms are very well supported by the model proposed by the authors in a wide range of temperatures and pressures. The proposed model takes into account the geometry of the adsorption sites; it was found that at low pressure, CO_2_ molecules adsorb with their long axis directed toward the center of the zeolite window, while at higher pressures, two oxygen atoms are equidistant from the Na^+^—ion at the bonding site [19].

The sorption of ammonia in FAU, MFI, and LTA silica zeolites was investigated by Matito-Martos et al. [20]. The authors measured the ammonia sorption isotherms at three temperatures of 258–298 K, due to the practical aspect of using the adsorption method to remove ammonia. The authors consider that the adsorption of ammonia in zeolites with large cavities is guided by the nucleation of these molecules, and it is possible due to the network of hydrogen bonds formed by the NH_3_ molecules [20].

The results of the presented study show the sorption of SO_2_, CO_2_, NH_3_ and H_2_O in two forms of faujasite and two forms of MFI zeolite. As the Si/Al ratio changes, the properties of zeolite, such as the acidity/basicity and hydrophilicity/hydrophobicity of the materials also change. The purpose of this study was to determine the adsorption affinity of the materials studied towards selected polar sorbates with different properties (structure, and chemical nature). The study of individual pairs also has a practical implication in predicting the efficiency of removal of these substances from mixtures. Due to the corrosive effect of ammonia and sulfur dioxide on the environment and research apparatus, there is a gap in the recent literature regarding the results of such studies.

Together with their good stability and availability (simple synthesis), these zeolites are interesting candidates for the capture of water vapor, ammonia, sulfur dioxide, and carbon dioxide via the pressure swing sorption processes.

## 2. Materials and Methods

### 2.1. Materials

The research was carried out on four samples belonging to two structural groups: faujasite (FAU) and Mobil-type five (MFI) zeolite.

Samples FAU (As-made) parent gel composition: SiO_2_:Al_2_O_3_:Na_2_O:H_2_O = 12.8:1:17:675—Z1, FAU (APrTMOS treatment)—Z2, MFI (Silicalite-1,) parent gel composition: SiO_2_:TPABr:H_2_O:NaOH: = 1:0.25:400.3—Z3, and ZSM-5 (TOSOH Corporation, Tokyo, Japan) Si/Al = 20—Z4, were the materials with selected zeolite structures chosen for the studies.

Faujasite has the most open framework of all natural zeolites [4,21]. The FAU unit cell contains eight cavities (the wide supercages) each of diameter about 1.3 nm. The interior of the supercage is accessed through 12-membered silicate rings with free apertures of about 0.74 nm. Each supercage consists of ten sodalite cages which are connected through hexagonal prisms. About half of the unit-cell space is void in the dehydrated form [22,23].

The second group—MFI (Mobil-type five) zeolites—form a three-dimensional system with a pore size of 0.5–0.6 nm [24]. The structure is a combination of two interconnected channel systems: sinusoidal 10-member-ring channels along the direction of the a-axis, and interconnected with 10-member-ring straight channels along the b-axis. Furthermore, the winding pore path is present along the c-axis [25]. Its surface area and pore volumes are about 400 g/m^2^ and 0.24 cm^3^/g, respectively.

FAU, described above, offers a higher surface area (above 700 m^2^/g) and a higher micropore volume (about 0.5 cm^3^/g) which generally leads to a higher sorption capacity compared to MFI [24].

Synthesis procedure:

In general, the Z1 preparation procedure involves mixing sodium silicate, sodium aluminate and water together and aging the resulting slurry at 313 K for a few days [26].

Z2 constitutes a modified variant of faujasite, wherein aminopropyltrimethoxysilane serves as a precursor for aminopropyl-substituted silica. Aminopropyltrimethoxysilane was hydrolyzed (H_2_O, EtOH, and NaOH) to deprotect the hydroxyl groups. This transformative process engenders the formation of a three-dimensional amorphous-modified silica species, intricately encapsulated within the zeolite (faujasite) matrix. The introduction of aminopropyltriethoxysilane into the zeolite backbone can give the material new properties. The resultant three-dimensional amorphous-modified silica not only retains the basic structural integrity of zeolite, but also exhibits additional functionalities due to the incorporation of aminopropyl-substituted silica moieties, such as greater potential for hydrogen bonding with the sorbent.

Z3 synthesis is based on the crystallization of silica on an ammonium salt (TPABr). The aqueous solutions with chemical compositions of SiO_2_:TPABr:H_2_O:NaOH = 1:0.25:40:0.3 led to silicalite-1 crystallization after hydrothermal treatment (453 K) [27].

### 2.2. Characterization Methods

#### 2.2.1. Helium Density

The helium density of the zeolites was measured with the AccuPyc Micrometrics 1330 device (Micromeritics Instrument Corporation, Norcross, GA, USA). The use of helium makes it possible to study porous media or media with an intrinsic surface structure. As a noble gas, helium does not interact with other substances, does not get adsorbed either at ambient temperature or at elevated temperatures, and its molecule is very small. That is why the use of helium allows to penetrate even the smallest pores, so the sample volume and density can be precisely determined.

The textural parameters (e.g., BET specific surface area and pore volume) were calculated from high-resolution nitrogen adsorption/desorption isotherms at 77 K, recorded using a volumetric analyzer 3Flex, (Micromeritics Instrument Corporation, Norcross, GA, USA) with a turbomolecular vacuum pump and three pressure transducers for accuracy in the low-pressure region. The samples were previously outgassed at 393 K for 12 h in vacuum.

#### 2.2.2. XRD Analysis 

X-ray measurements were performed using a PANalytical Empyrean powder diffractometer with Bragg–Brentano geometry (Malvern, United Kingdom). The diffractometer was equipped with a copper anode lamp with a wavelength of λ = 0.154187 nm. Measurements range from 4 to 50 degrees. The use of X-ray diffraction allowed us to confirm the crystal structure of the studied zeolites. The diffractograms were processed in OriginPro 2022.

#### 2.2.3. FTIR Analysis

Infrared analysis of the materials was carried out using a PerkinElmer Frontier FT-IR spectrometer (Waltham, MA, USA). Analyses were performed in transmittance mode in a wavenumber range 4000 to 400 cm^−1^, in KBr. The spectra were processed in SpectraGryph 1.2.15.

#### 2.2.4. Sorption Experiments

Sulfur dioxide sorption experiments were performed using the weight method, while carbon dioxide, ammonia, and water sorption were tested using the volumetric method. Samples were degassed before measurement. The SO_2_, CO_2_ and NH_3_ measurements were carried out at a temperature of 25 °C, while the water sorption was carried out at 30 °C. The samples were degassed (up to a pressure of 10^−5^ bar) and rinsed with helium (for 24 h) before measurement [13,28,29].

#### 2.2.5. SEM Analysis 

Scanning electron microscopy analysis–energy dispersive X-ray spectroscopy (SEM/EDS) was performed with the aid of JEOL JSM-7500F, coupled with an AZtecLiveLite Xplore 30 (Oxford Instruments, Oxford, UK) system. The secondary electron detector provided SEI images and the backscattered electron detector provided BSE (COMPO) micrographs. SEM images were recorded for samples coated with 30 nm Cr. SEM (Scanning Electron Microscopy) makes use of secondary electron signal imaging to observe the surface morphology of the sample, to infer material components, and to reveal the microstructure on the micro and nanometer scale.

## 3. Results

### 3.1. Textural Properties and Porosity

Measurements were carried out using low-pressure nitrogen sorption. The following textural parameters were determined from N_2_ adsorption–desorption isotherms at 77 K:

S_BET_ [m^2^/g]—specific surface area using Brunauer–Emmett–Teller (BET) analysis, V_DR_ [cm^3^/g]—volume of micropores from Dubinin–Radushkevich equation, S_DR_ [m^2^/g]—surface area of micropores from Dubinin–Radushkevich equation, V_BJH_ [cm^3^/g]—volume of mesopores from Barrett–Joyner–Halenda (BJH) analysis, S_BJH_ [m^2^/g]—surface area of mesopores Barrett–Joyner–Halenda (BJH) analysis, and V_total_ [cm^3^/g]—total pore volume calculated at p/p_0_ = 0.995. The compared parameters are summarized in Table 1.

All tested materials differ in terms of micropore volume and specific surface area. Z1 has the largest specific surface area (816.5 m^2^/g), while Z2 has the smallest (38.9 m^2^/g). The 20-fold reduction in the surface area of sample Z2 was due to the reduction in the number of pores available through silane modification.

Samples Z3 and Z4 have a specific surface area twice as small as faujasite (358.0 and 416.4 m^2^/g, respectively) and this is a characteristic value for MFI zeolites.

### 3.2. XRD Analysis

**Figure 1:**
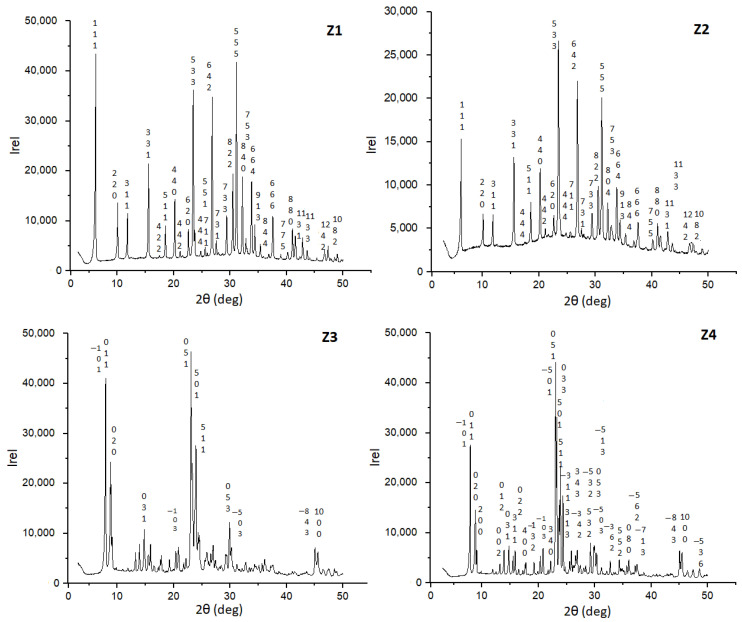
XRD patterns of materials Z1–Z4.

Figure 1 shows the XRD patterns of all samples. XRD patterns of Z1 and Z2 correspond to the crystalline structure of faujasite according to the tables of the International Zeolite Association [30]. The amine does not affect the crystal structure of the modified faujasite, as shown in Figure 1.

Similarly, the Z4 diffraction pattern corresponds to the crystalline structure of ZSM-5 [25]. The diffraction peaks of Z3 (2θ = 7.95°, 8.86°, 14.00°, 14.85°, 23.00°, 23.90°, and 24.00°) correspond to the characteristic peaks of the MFI zeolites [30,31].

### 3.3. FTIR Analysis of Materials before Sorption Experiments

**Figure 2:**
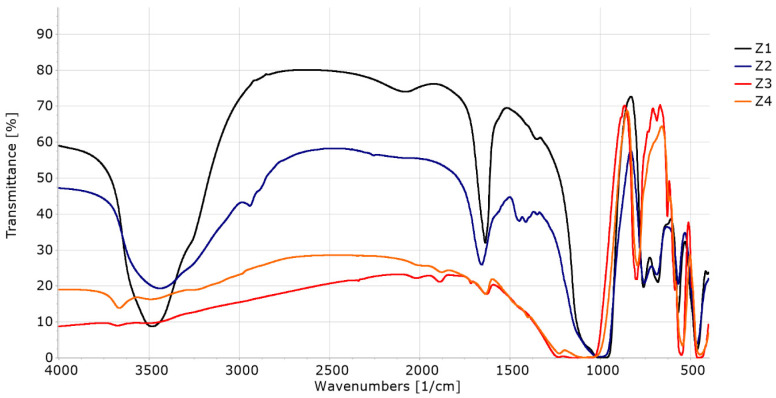
FTIR spectra of materials Z1–Z4.

The spectra of all samples (Z1–Z4) are presented in Figure 2. In both samples of faujasite (Z1 and Z2), the bands at 3750 to 3450 cm^−1^ are attributed to Si-OH, Si-OH-Al, and –OH hydroxyl groups. The bands at 1200 to 450 cm^−1^ are known to assign to Si-O-Al, Si-O-Si, Si-O, and Si-Al. The band at 660 cm^−1^ is known to assign to Si-O m, where M is the exchangeable Na^+^—ion metal species. The bands around 1600 cm^−1^ come from H-O-H vibration and indicate the presence of water bound in the structure [32]. In the infrared spectrum of the Z2 sample (APrTMOS treatment), we can also observe the broadening of the signal from the amine groups (3600–3200 cm^−1^), C-H aliphatic vibrations (2940–2880 cm^−1^) and the broadening of the signal at 1250–900 cm^−1^ (Si-O-C and Si-C-C vibrations) compared to faujasite (Z1) [33]. The spectra confirm the faujasite structure for Z1 and Z2. 

Z3 and Z4 represent a different type of structure (MFI), which is clearly manifested in their IR spectra. These materials have a strong absorbance of infrared radiation in the range of 4000–1000 cm^−1^, which is a characteristic feature of MFI zeolites.

### 3.4. SEM Analysis

**Figure 3:**
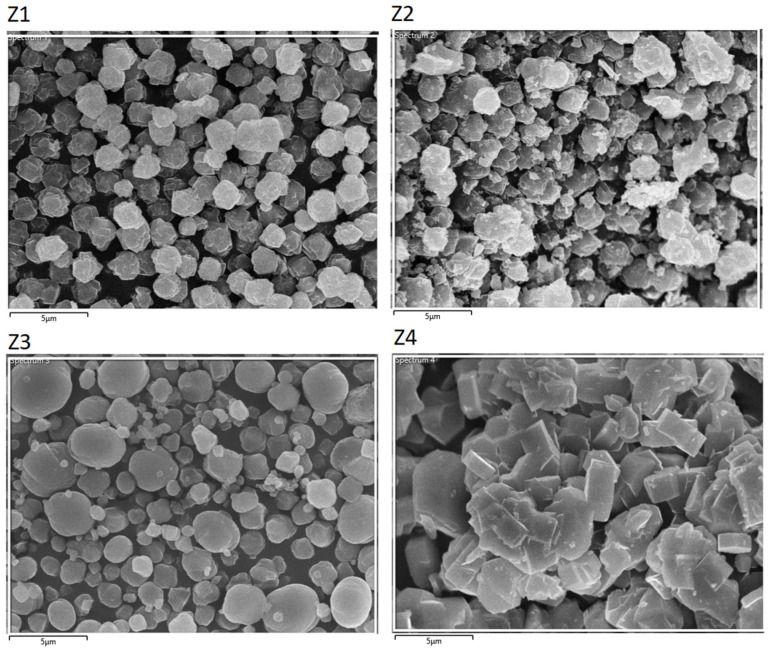
SEM photos of materials (**Z1**–**Z4**).

Figure 3 shows SEM images for all the materials (Z1–Z4) and Table 2 compares the results of EDX analysis. Each of the samples has a slightly different grain morphology.

The common feature of images Z1 and Z2 is the dominant presence of grains composed of irregular shaped plates. In the case of Z2, these plates coexist with irregular particles of various dimensions (1–3 µm). Z1 has grains of similar size (2 µm).

Z3 features the presence of spheroidal-shaped grains of different sizes (1–4 µm). Higher magnification reveals that all grains are made up of tightly packed plates with rounded edges. The thickness of these plates appears to be less than that of Z1 and Z2.

Z4 is built of the biggest grains composed of much thicker and larger plates. In contrast to Z3, the well-shaped plates feature sharp edges. The grain size distribution is also large (2–5 µm).

### 3.5. Adsorption Isotherms

**Figure 4:**
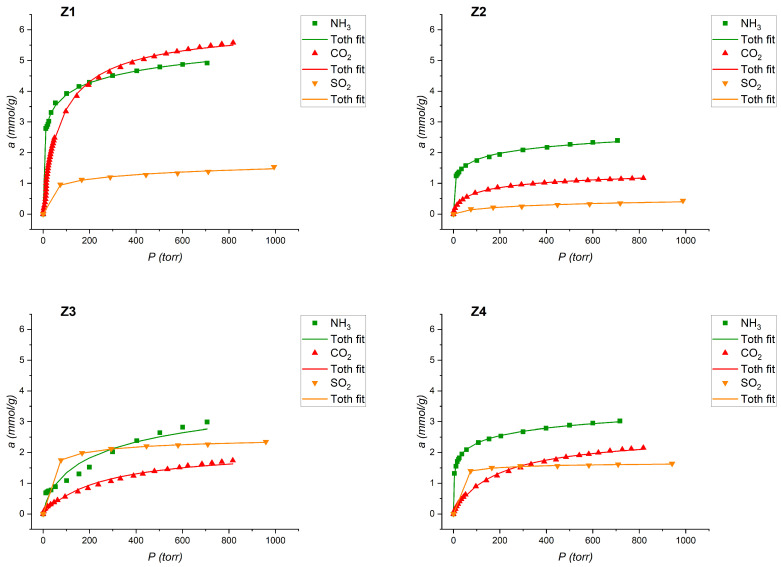
(**Z1**–**Z4**) adsorption isotherms (T = 298 K) for NH_3_, SO_2_, and CO_2_ and its compatibility with the Toth model.

**Figure 5:**
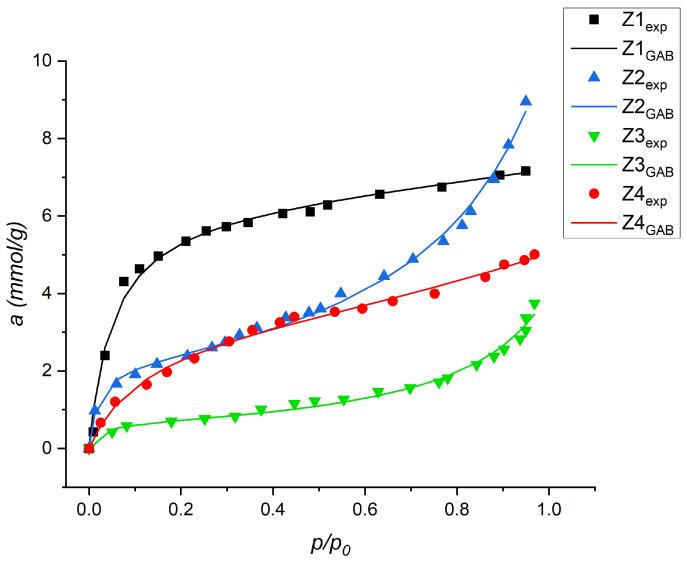
Z1–Z4 adsorption isotherms (T = 303 K) for H_2_O and its compatibility with the GAB model.

The sorption isotherms of NH_3_, SO_2_, and CO_2_ were measured at 298 K and are shown collectively in Figure 4. The vapor pressure of water at a temperature of 303 K is in a different pressure range compared to the sorption conditions of the rest of sorbates, therefore, the diagram is presented separately (Figure 5).

Experimental data (dots) were fitted to Langmuir, Toth, GAB and Dubinin–Radushkevich models, and they showed the best fit for the Toth model (for NH_3_, SO_2_, and CO_2_) and the GAB model (for H_2_O) (solid line) [13,34,35].

The Toth isotherm is an empirical modification of the Langmuir equation with the intent of reducing the error between the experimental data and the predicted value of the equilibrium data. The Toth isotherm model is often used to describe adsorption on heterogeneous surfaces by introducing the parameter *t*. With this additional parameter, Toth’s equation can accurately describe a large number of adsorbent–adsorbate systems, which include NH_3_, SO_2_ and CO_2_ on activated carbons and zeolites. Toth isotherm is expressed as follows:

(1) $$n\left(p\right)=n_{m}\frac{Kp}{\sqrt[{t}]{1+{\left(Kp\right)}^{t}}}$$

where, *n_m_* is the monolayer capacity, 𝑝 is pressure, *K* is the Toth model constant, and *t* is parameter which describes the heterogeneity of the adsorption system; the more it deviates from unity, the more heterogeneous the system is. When *t* is equal to one, the equation reduces to the Langmuir isotherm [36].

The Guggenheim–Anderson-de Boer (GAB) is the most common model used to fit the adsorption and desorption isotherms of water vapors for different materials. It is divided into two additive terms: the first one describes the classical monomolecular layer expression in Langmuir’s adsorption isotherms, and the second term describes the multilayer adsorption corresponding to Raoult’s law. This model postulates that the state of sorbate molecules in the second layer is identical to that in the superior layers but different from those of the liquid state. It describes the sorption behavior in a wide range of relative pressure (*p/p*_0_ = 0.1–0.9).

The GAB model is expressed as follows:

(2) $$n\left(p\right)=n_{m}\frac{CKp}{\left(1-Kp\right)(1-Kp+CKp)}$$

where, *n_m_* is the monolayer capacity, 𝑝 is pressure, *C* is the kinetic constant related to the sorption in the first layer, and *K* is the kinetic constant related to multilayer sorption. *C* and *K* are the adsorption constants considering the different enthalpy of adsorption of the adsorbed phase compared to the enthalpy of condensation of the bulk phase [37,38,39].

Z1 showed the best sorption properties for ammonia, water, and carbon dioxide. Polar interactions of ammonia and water with surface groups result in a relatively high sorption capacity [40,41]. 

The MFI Z3 shows the highest sorption properties of sulfur dioxide, but this value differs only slightly from the other samples. This experiment was carried out under dry and anaerobic conditions, but it is not clear whether there was only physical adsorption of SO_2_. This is confirmed by the baring of the materials after sorption measurements.

Z2 shows a low sorption capacity for all sorbates, except water. Amine functionalization of zeolites and other materials with sorption properties has been described many times in the context of carbon dioxide adsorption [42,43,44,45,46]. Unfortunately, the preparation of the sample with the use of silane as a silica precursor did not result in positive effects in the form of a large development of the specific surface. However, we observed a significant increase in the sorption capacity of water vapor. The shape of the adsorption curve indicates strong capillary condensation of water in the Z2 mesopores. We believe this effect is due to the formation of hydrogen bonds between the amino groups deposited on the surface of the zeolite and the water molecules.

All the descriptors have been completed and the individual affinity of the gases towards the inorganic materials studied is evident. The sorption capacity corresponds to the textural parameters of the inorganic sorbents (Table 1) through the molecular sieve effect. In samples Z3 and Z4, despite having a relatively high specific surface area compared to Z1 and Z2 materials, adsorption occurs in a porous system. In contrast, sample Z2 (containing amine groups) with a very low specific surface area sorbs similar amounts of all tested sorbates as Z3 and Z4. This indicates the dominant contribution of polar interactions of sorbates with the functional groups of the surface of sample Z2.

In the case of sample Z3, the shape of the ammonia isotherm significantly differs from the other adsorption materials, due to which high NH_3_ sorption capacity was found already at low relative pressures. Moreover, the value of sorption capacity in Z3 is comparable with samples Z2 and Z4; nevertheless, the increase in absorptivity is proportional to pressure. Thus, it can be concluded that in this case there was a change in the density of the adsorbed phase, which changes with increasing pressure.

### 3.6. FTIR Analysis of Materials after Sorption Experiments

**Figure 6:**
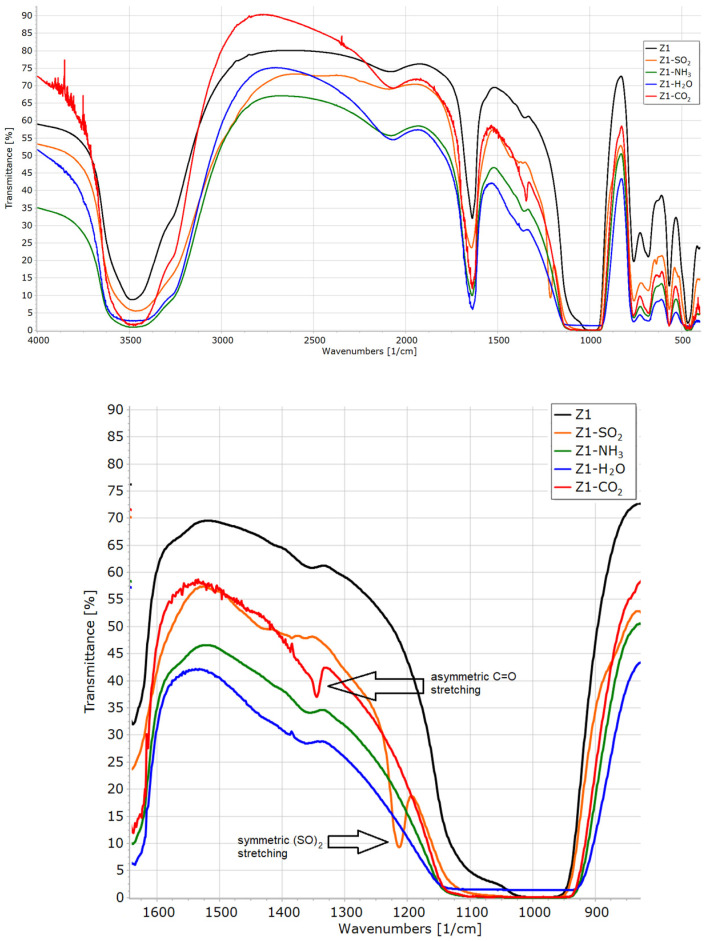
FTIR spectra of Z1 before and after sorption experiments.

**Figure 7:**
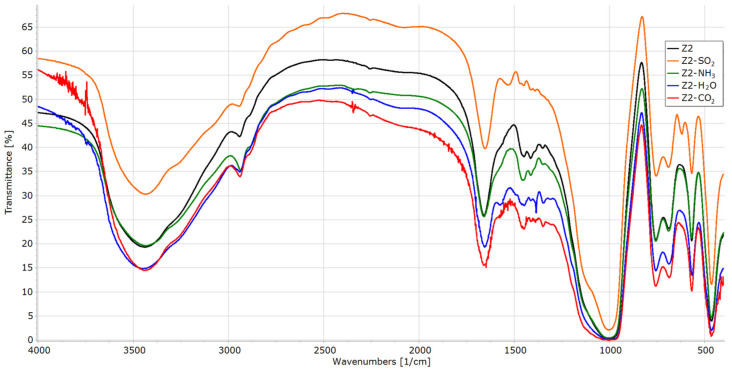
FTIR spectra of Z2 before and after sorption experiments.

**Figure 8:**
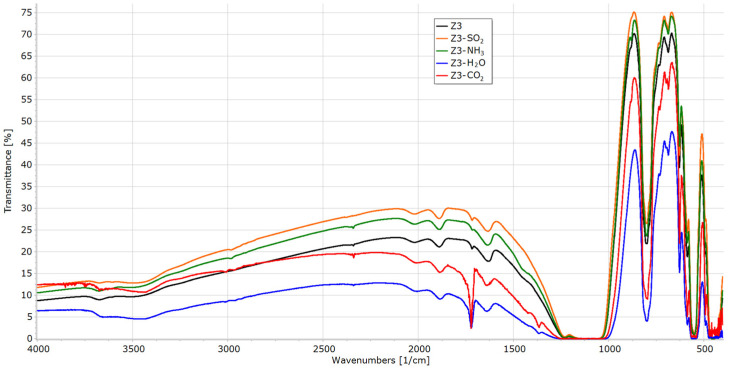
FTIR spectra of Z3 before and after sorption experiments.

**Figure 9:**
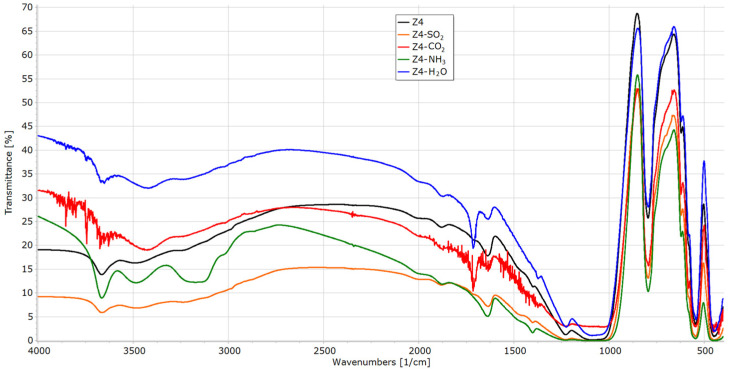
FTIR spectra of Z4 before and after sorption experiments.

All samples after sorption experiments were re-examined with a FTIR spectrometer (Figure 6, Figure 7, Figure 8 and Figure 9).

In the infrared spectrum of an unmodified Z1 after SO_2_ sorption, we can observe the sharp signal at 1213 cm^−1^, characteristic of symmetric (S=O) stretch [30,47]. Ammonia and water sorption are visible in the spectrum as a bordering of the signal in the range of 3700–3150 cm^−1^. The bands at 3960–3730 cm^−1^ (combination band) and 2350 cm^−1^ (asymmetric C=O stretching) indicate the physical adsorption of carbon dioxide. A band at 1345 cm^−1^ may indicate the formation of carbonate-like species (chemisorption) [48].

In the case of the Z2 sample, the signal from SO_2_ is not visible as it is superimposed with a broad signal from Si-O-C and Si-C-C vibrations (1250–900 cm^−1^). Furthermore, this material is the weakest SO_2_ adsorbent. The signal 3700–3150 cm^−1^ is slightly broadened after an ammonia sorption and even more after a water sorption. The bands at 3950–3730 cm^−1^ (combination band) and 2348 cm^−1^ (asymmetric C=O stretching) indicate the physical adsorption of carbon dioxide.

In a spectrum of silicate-1 (Z3, Figure 9), the signal from SO_2_ is not visible as it overlaps with the spectrum of the parent sample. The ammonia and water adsorption signals are weak; however, this material adsorbed in a much worse way compared to those of both faujasites (Z1 and Z2). The band at 1723 cm^−1^ comes from the H-O-H vibration and indicates the presence of water adsorbed by the material. Bands at 3950–3800 cm^−1^ (combination band) and 2340 cm^−1^ (asymmetric C=O stretching) indicating CO_2_ sorption are less visible, which correlates with the low sorption capacity of this gas.

In a spectrum of ZSM-5 (Z4, Figure 9), the signal from SO_2_ is not visible, as it overlaps with the spectrum of the parent sample. The band at 1718 cm^−1^ comes from H-O-H vibration and indicates the presence of water adsorbed by the sample. The bands at 3940–3600 cm^−1^ (combination band) and 2350 cm^−1^ (asymmetric C=O stretching) indicate CO_2_ sorption. The broad band from 3560 to 3350 cm^−1^ represents the asymmetrical and symmetrical N-H stretching modes of sorbed ammonia. The 3670 cm^−1^ band comes from hydrogen-bonded silanol (with NH_3_) silanol from the framework of the inorganic material [49].

## 4. Discussion

All skeletons were considered rigid because it was assumed that the influence of zeolite elasticity on the sorption of small molecules at the tested temperatures was small [50]. In the case of sulfur dioxide adsorption, one should expect dominant interactions with chemical groups present on the surface of the adsorbents. The microporous system does not play a dominant role here, which is confirmed by the isotherm results, where the sorption capacity of sample Z1 (with by far the highest specific surface area) of sulfur dioxide is slightly higher than for the other samples. In the case of SO_2_ adsorption, the dominant interaction is the formation of hydrogen bonds with functional groups present on the surface. The work of Verner et al. [51] demonstrated a high affinity of SO_2_ for the surface of various adsorbents (activated carbon, bentonite, and silica gel) which was interpreted by both the porosity and the chemical nature of the surface of these materials.

The sorption of ammonia (and probably water) in pure silicate zeolites is regulated by the nucleation of this molecule inside large cavities. Ammonia (and water) on the surface of the adsorbent can behave like a liquid and form a network of hydrogen bonds, which is slightly weakened by encapsulating in the zeolite cavities [19].

However, as a result of the reactivity of ammonia, a small amount of off-frame cations can have a noticeable effect on adsorption. The surface properties of the zeolite, such as acidity/alkalinity and hydrophilicity/hydrophobicity can vary depending on the Si/Al ratio. Zeolite-based membranes have shown great potential for separating xylenes [52,53], and more recently for separating mixtures containing ammonia, nitrogen and hydrogen [54,55]. Adsorption and diffusion processes are also being studied in fully periodic MFI zeolite crystals and in a more complex 3 nm thick MFI zeolite nanoshell model, which contains surface silanol groups (Si-O-H) that can interact strongly with ammonia [56]. The adsorbent and adsorbate may undergo phase transformations that would explain the experimental observations. Condensation of ammonia within the pores of zeolite is possible as a consequence of entrapment. Another explanation could be the structural phase transition of MFI after ammonia adsorption [54,55]. The increase in the amount adsorbed at high relative pressures is not attributed to the phase transition, but rather to the nucleation of the fluid inside the large cavities of this zeolite. The shape of the isotherm may also be related to the higher mobility of the adsorbate at the temperature of the experiment, which would reduce its density and thus reduce the interaction of ammonia with the zeolite walls. Unfortunately, such a phase transition is a rare phenomenon that is not sufficiently described in the literature. FAU (1), on the other hand, is a zeolite with a high silica content (Si/Al ratio of 22), which means that it contains a small (but not negligible) amount of off-node protons (www.zeolyst.com, accessed on 20 March 2023). This slightly modifies the electrostatic field inside the zeolite pores. This fact does not affect the adsorption of small gases or other nonpolar molecules. However, ammonia is a very reactive compound that can strongly interact with impurities or extralayer cations present in zeolites. Ammonia molecules that react with protons produce ammonium cations (NH_4_^+^), where the newly formed covalent bond is indistinguishable from the other NH bonds. Deciphering the mechanism of the reaction [57] is beyond the scope of this work; however, a simplification can be performed to investigate the effect of possible ammonium cations of ammonium cations formed during the adsorption process.

The results of water vapor sorption isotherms remain in relation with those for the other sorbates, especially ammonia. In the case of sample Z1, we observe the classical effect of water vapor sorption in the area of the microporous structure. The high specific surface area of this sample correlates with the shape of the type I isotherm according to the IUPAC. In the case of sample Z2, the shape of the isotherm (type II according to the IUPAC) indicates the formation of a multi-molecular adsorption layer of water molecules.

Water vapor is considered a probe for assessing the chemical nature of the adsorbent, (in this case, the low specific surface area does not coincide with the high adsorption value, indicating condensation of H_2_ vapor) at higher relative pressures. This provides confirmation of strong interactions of water molecules with silane groups.

The high separation selectivity is attributed to the narrow channel with lined amino groups, which exhibits significantly higher affinity for SO_2_ than CO_2_.

## 5. Conclusions

Research has shown that faujasite has the best sorption capacity for ammonia, water vapor, and carbon dioxide, while the sorption capacity of sulfur dioxide is similar for all four zeolites. 

Compared to Z1, Z2–Z4 show similar, and much lower ammonia sorption capacity (approx. 4.7 mol/kg vs. 2.0–2.5 mol/kg) in the tested pressure range. In the literature, the sorption of ammonia in zeolites with large cavities is regulated by the nucleation of these molecules. This is because ammonia can form a network of hydrogen bonds similar to those found in water or light alcohols [19].

Treatment of the faujasite with an organic amine—aminopropyltrimethoxysilane—caused a dramatic decrease in the surface of the sorbent. Since the size of the pores has not changed, their number must have decreased; therefore, it is likely that the coating of faujasite with silane caused the clogging of most of the pores, as a result of which the gases sorption capacities were significantly lower. Only the sorption of water vapors was favorable, which may be related to the formation of hydrogen bonds between water molecules and the—NH_2_ group of aminosilane.

The relatively high capacity, stability, and commercial availability make faujasite (Z1) an interesting candidate for the capture of ammonia, water vapor, and carbon dioxide in the pressure swing sorption processes.

## Figures and Tables

**Table 1:** Textural properties of the zeolites.

	Z1	Z2	Z3	Z4
S_BET_ [m^2^/g]	816.5	38.9	358.0	416.4
V_DR_ [cm^3^/g]	0.310	0.015	0.137	0.152
S_DR_ [m^2^/g]	926.7	39.2	410.3	455.3
V_BJH_ [cm^3^/g]	0.024	0.021	0.081	0.065
S_BJH_ [m^2^/g]	17.4	17.5	100.3	65.3
V_total_ [cm^3^/g]	0.333	0.035	0.194	0.217
Density [g/cm^3^]	2.059	2.098	2.465	2.339

**Table 2:** EDX analysis.

	O	Si	Al	Na
	wt.%			
Z1	51	21	15	13
Z2	46	31	14	9
Z3	58	41	1	0
Z4	56	42	2	0

## Data Availability

The data presented in this study are available on request from the corresponding author. The data are not publicly available due to data privacy.
